# Baseline Susceptibility of Filarial Vector *Culex quinquefasciatus* (Diptera: Culicidae) to Five Insecticides with Different Modes of Action in Southeast of Iran

**Published:** 2017-12-30

**Authors:** Yaser Salim-Abadi, Mohammad Asadpour, Iraj Sharifi, Alireza Sanei-Dehkordi, Mohammad Amin Gorouhi, Azim Paksa, Zohre Tayyebi, Abbas Aghaei-Afshar

**Affiliations:** 1 Department of Health Services and Health Promotion, School of Health, Rafsanjan University of Medical Sciences, Rafsanjan, Iran; 2 Pistachio Safety Research Center, Rafsanjan University of Medical Sciences, Rafsanjan, Iran; 3 Leishmaniasis Research Center, Kerman University of Medical Sciences, Kerman, Iran; 4 Department of Medical Entomology and Vector Control, Faculty of Health, Hormozgan University of Medical Sciences, Bandar Abbas, Iran; 5 Infectious and Tropical Diseases Research Center, Hormozgan Health Institute, Hormozgan University of Medical Sciences, Bandar Abbas, Iran; 6 Department of Medical Entomology and Vector Control, School of Health, Kerman University of Medical Sciences, Kerman, Iran; 7 Department of Medical Entomology and Vector Control, School of Public Health, Tehran University of Medical Sciences, Tehran, Iran; 8 Student Research Committee, Rafsanjan University of Medical Sciences, Rafsanjan, Iran

**Keywords:** Susceptibility status, Resistance, Insecticide, *Culex quinquefasciatus*, Iran

## Abstract

**Background:**

*Culex quinquefasciatus* (Diptera: Culicidae) is an important vector for many human diseases. The aim of this study was to evaluate the susceptibility level of larval and adult stages of *Cu. quinquefasciatus* to different groups of WHO recommended insecticides for vector control.

**Methods:**

Larval stages of the *Culex* mosquitoes were collected from their natural habitats in Rafsanjan County at Kerman Province, southeast of Iran in 2016. Insecticide susceptibility status of adult female *Cx. quinquefasciatus* against DDT (4%), deltamethrin (0.05%), malathion 5%, and bendiocarb (0.1%) were determined using WHO standard insecticide susceptibility test. Additional test was carried out to determine the susceptibility status of larvae of *Cx. quinquefasciatus* to temephos. Bioassay data were analyzed by Probit program.

**Results:**

*Cx. quinquefasciatus* adults showed resistance to all four groups of the tested insecticides according to the WHO criteria for resistance evaluation. The lethal concentrations for 50% mortality (LC_50_) and 90% mortality (LC_90_) of temephos against *Cx. quinquefasciatus* larvae were 0.18mg/l and 0.78mg/l, respectively. This finding also confirms resistance to temephos based on the WHO recommended instructions for resistance evaluation.

**Conclusion:**

Resistance to all groups of the tested insecticides should be considered for future vector control investigations in the study area.

## Introduction

The southern house mosquito, *Culex quinquefasciatus* (Diptera: Culicidae) is an important vector for many human diseases. This species plays a crucial role in the transmission of some important pathogen such as *Wuchereria bancrofti*, *Dirofilaria immitis*, *Plasmodium relictum*, Sindbis virus, West Nile virus, Equine encephalitis, St Louis, Oropouche and Rift Valley fever which are today among the major public health problems worldwide (1–8).

Wastewater and sewage system are important breeding places for *Culex* mosquitoes. Constant exposure of *Cx. quinquefasciatus* to the high organic content of wastewater including detergents, different groups of insecticides, industrial pollutants, and oil compounds can lead to the development of resistance in mosquito larvae against insecticides and larvicides (9–11). In recent years, increasing level of resistance to various groups of insecticides has been a major barrier to the success of vector control programs. Many studies have reported high level of resistance in *Cx. quinquefasciatus* to many groups of insecticides (12–14). *Culex quinquefasciatus* is an important member of *Cx. pipiens* complex wildly distributed worldwide (2, 5, 15).

In Iran, the resistance status of *Cx. pipiens* complex against different groups of insecticides was indicated the development of resistance in the members of this species, including *Cx. quinquefasciatus*, during the past quarter-century: development of resistance to most of the organochlorine insecticides including DDT (Dichloro diphenyl trichloroethane) (6, 11, 16–20). Resistance to pyrethroid insecticides such as lambda-cyhalothrin, deltamethrin, and cyfluthrin (6, 16, 17, 19). Resistance to the carbamate insecticides propoxur and bendiocarb (11, 16, 17) and relative resistance to malathion organophosphates insecticides (11, 16). Moreover, resistance of the larvae of *Cx. pipiens* complex to temephos has recently been reported for the first time in Iran (9).

There was no study on monitoring the susceptibility level of *Cx. quinquefasciatus* to insecticides in Rafsanjan County at Kerman Province, southeastern Iran. We aimed to determine the susceptibility status of *Cx. quinquefasciatus* against insecticides in this area.

## Materials and Methods

### Study area

**Fig. 1.**
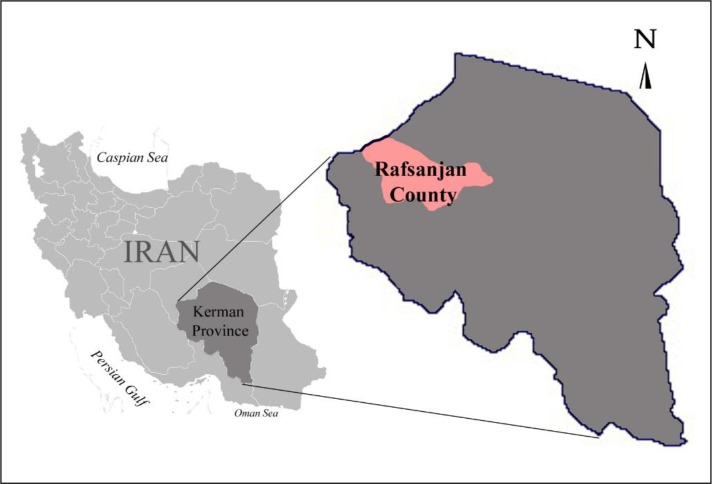
The geographical location of Rafsanjan County in Kerman Province, Iran

This study was carried out in Rafsanjan County at Kerman Province, southeastern Iran. The county located at latitude 30°30′N and longitude 55°40′E, with a population of 300000 in 2015 (Fig. 1).

### Bioassay procedure

Larvae of *Cx. quinquefasciatus* were collected from larval habitats in Rafsanjan County in 2016, and all sample were transferred to laboratory and reared at 27 °C and 65±5% relative humidity using a 12h light/12h dark photoperiod. Bioassay tests were carried out using WHO test kits on adult mosquitoes (21). The following diagnostic concentrations of insecticides were tested: DDT 4%, lambda-cyhalothrin 0.05%, malathion 5%, and bendiocarb 0.1%. Tests were carried out on3 to 5-day-old unfed females. Batches of 25 females were exposed to insecticide-impregnated papers at different exposure times. Two replicates of 25 adult mosquitoes (3 to 5-day-old unfed females) were considered as controls with untreated papers for each different exposure time. The lethal time for 50% mortality and 90% mortality (LT_50_ and LT_90_) among the mosquitoes was calculated using log-probit software according to Finney’s formula (22, 23). Larvicide susceptibility tests were carried out on late 3^rd^ to early 4^th^ stage larvae to determine larval susceptibility to temephos using WHO standard kit (1.25, 6.25, 31.25 and 156.25 mg/l concentrations), according to WHO instructions (22, 23). Each test consisted of four replicates with 25 larvae each in glass beakers containing 250ml of distilled water and the specified insecticide concentration. Two replicates of 25 untreated larvae were maintained as controls. After 24h exposure period, larval mortality was calculated the lethal concentrations for 50% mortality and 90% mortality (LC_50_ and LC_90_) were calculated by probit analysis (24). In both adult and larval susceptibility testing, mortality rate in the test samples was corrected using Abbott formula (25), when the mortality rate of control was between 5% and 20%.

## Results

The mortality rate (MR) in adult *Cx. quinquefasciatus* mosquitoes exposed to four different groups of insecticide-impregnated papers are shown in Table 1 and 2. Lambda-cyhalothrin with LT_50_= 25 minute and LT_90_= 74min and DDT with LT_50_= 139min and LT_90_= 227min had the lowest and highest LT_50_ and LT_90_ values, respectively (Table 1).

**Table 1.** Probit regression line parameters of *Culex quinquefasciatus* exposed to different groups of insecticides in Rafsanjan City, southeastern Iran, 2016

**Insecticides**	**A**	**B ± SE**	**LT_50_, 95% C.I. (Min)**	**LT_90_, 95% C.I. (Min)**	**X^2^ (df)**	**p value**
**Lambda-cyhalothrin 0.05%**	−3.80	2.72 ± 0.27	22	58	5.84(2)	>0.05
			25	74		
			29	102		
**Malathion 5%**	−3.89	2.55 ± 0.28	29	80	5.7 (2)	>0.05
			33	106		
			39	159		
**Bendiocarb 0.1%**	−3.90	2.72 ± 0.28	23	63	5.49 (2)	>0.05
			27	79		
			31	112		
**DDT 4%**	−13.01	6.06 ± 0.55	129	204	3.04 (2)	>0.05
			139	227		
			172	260		

A= y-intercept, B= the slope of the line, SE= standard error, CI= confidence interval, x^2^ = heterogeneity about the regression line, df= degree of freedom, P> 0.05= represents no heterogeneity in the population of tested mosquitos.

**Table 2.** Susceptibility level of *Culex quinquefasciatus* exposed to different groups of insecticides in Rafsanjan County, southeastern Iran, 2016

**Insecticides**	MR ± EB^*^	Resistance status^**^
**Lambda-cyhalothrin 0.05%**	90 ± 2	RC
**Malathion 5%**	80 ± 3	R
**Bendiocarb 0.1%**	88 ± 3	R
DDT 4%^***^	90±2	RC

* Mortality rate± errorbar

** RC Resistance Candidate

*** After 4 h exposure period

**Fig. 2.**
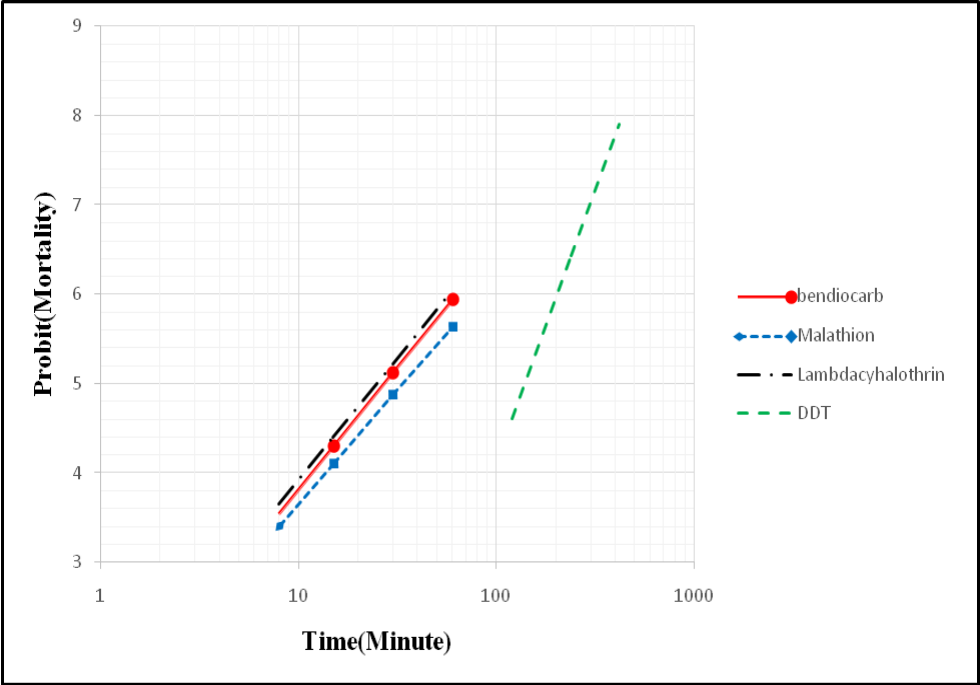
Regression lines of *Culex quinquefasciatus* exposed to different group of insecticides in Rafsanjan City, Southeastern Iran, 2016

*Culex quinquefasciatus* is resistant to malathion and bendiocarb and candidate of resistance to lambda-cyhalothrin and DDT based on the criteria for insecticide resistance described by WHO (Table 2). The mortality rate in the mosquitoes at one-hour exposure to the insecticides calculated after 24h recovery period has been summarized in Table 2. Malathion had a mortality rate of 80% (MR= 80%), bendiocarb 88%, lambda-cyhalothrin and DDT 90% each, the mortality rate of DDT was calculated after 4h exposure time instead of 1h (Table 2). The regression line of different concentration is shown in Fig. 2.

**Fig. 3.**
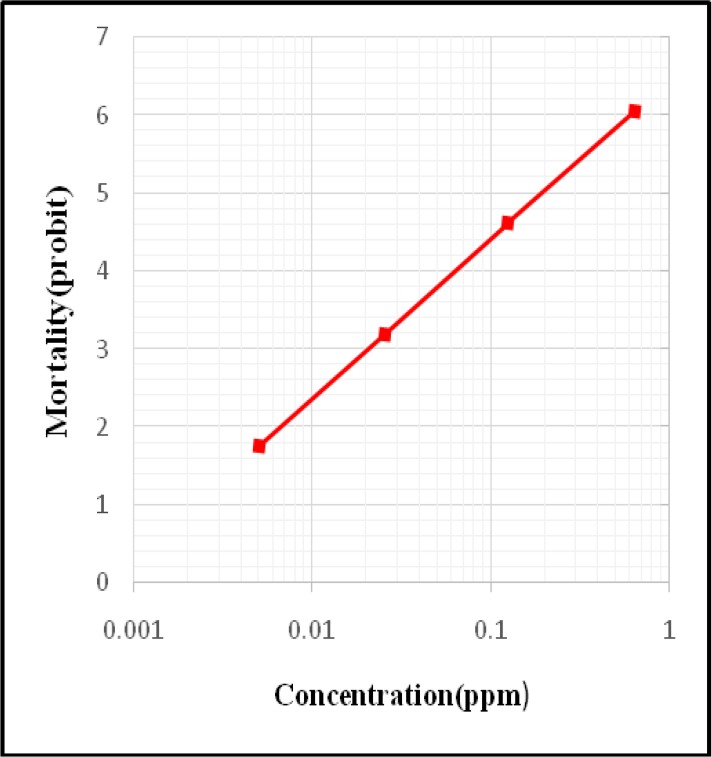
Mortality Regression lines of different concentrations of Temephos against *Culex quinquefasciatus* in Rafsanjan City, Southeastern Iran, 2016

The mortality rate of each concentration of temephos is shown in Table 3. The mortality rate of temephos ranged from 3% to 100%. Moreover, using Finney’s method, we calculated LC_50_ and LC_90_ for *Cx. quinquefasciatus* that were 0.18 and 0.78 ppm, respectively (Table 4). The regression line of the different concentrations of temephos is shown in Fig. 3.

**Table 3.** Mortality rate in *Culex quinquefasciatus* larvae at WHO standard concentrations of Temephos in Rafsanjan County, Southeastern Iran, 2016

**Concentration (ppm)**	**Replicates**	**No. of tested larvae**	**No. of mortality**	**Mortality rate (%)**	**Observed mortality probit**	**Expected mortality probit**
**0.005**	4	100	3	3	3.119	1.762
**0.025**	4	100	6	6	3.445	3.199
**0.125**	4	100	10	10	3.718	4.636
**0.625**	4	100	100	100	7.576	6.073
**Control**	2	50	0	0	-	-

**Table 4.** Probit regression line parameters of Temephos against *Culex quinquefasciatus* larvae in Rafsanjan County, Southeastern Iran, 2016

**A**	**B**	**LC_50_, 95% CI (ppm)**	**LC_90_, 95% CI (ppm)**	**X^2^ (df)**	**P-value**
1.49	2.05	0.18	0.78	190.76(2)	<0.05

## Discussion

The present study provides evidence of resistance to four different classes of insecticides according to the current WHO criteria for insecticide resistance evaluation.

The mortality rate was interpreted as follows: higher than 98% was considered as susceptible, less than 90% indicated resistance, and from 90% to 97% was defined as resistance candidate. For the resistance candidate category (90–97% mortality rate), additional investigation is needed for the confirmation of resistance (21). Although both lambda-cyhalothrin and DDT have mortality rate of 90% each which classifies them as potential candidate for resistance according to the WHO criteria.

This finding can be useful in future vector control programs and investigations in order to prevent the development of resistance to both insecticides.

Previous studies have also reported *Cx. pipiens* resistance to different classes of insecticides in different geographical regions of Iran, resistance to DDT, lambda-cyhalothrin, deltamethrin and cyfluthrin in Tehran, capital of Iran (6, 11, 20). Resistance to DDT in the North of Iran (18), resistance to DDT, lambda-cyhalothrin and propoxur in a dirofilariasis foci in the Northwest of Iran (18), resistance to DDT, propoxur, cyfluthrin and lambda-cyhalothrin and tolerance to deltamethrin in a malaria endemic area in the Southeastern part of Iran (17), and resistance to deltamethrin and DDT in the Northwestern part of Iran (19).

The use of pesticides in agricultural sector can lead to the development of resistance to insecticides in medically important vectors including *Cx. quinquefasciatus* (13, 26–28).

There is a growing concern over the development of multiple insecticide resistance mechanisms in medically important arthropods that is a major problem in vector control (15, 29–31). Over the last fifty years, resistance to insecticides has been a growing concern. Resistance of mosquitoes to DDT was first reported in 1949 (32, 33). However, resistance to organophosphorus insecticides in *Cx. quinquefasciatus* was first reported in 1961 (34), and to date, there have been several reports on resistance to various classes of insecticides in *Cx. quinquefasciatus.* This species is now quite resistant to some insecticides such as DDT and Malathion such that it does not exhibit mortality at one-hour exposure and 24h recovery period (35).

In the present study, in addition to adult susceptibility test, susceptibility of the larvae of *Cx. quinquefasciatus* to Temephos was evaluated according to WHO standard method. We observed mortality rate ranged between 3% and 100%. In another study, laboratory evaluation of the susceptibility of *Anopheles stephensi* larvae collected from Kazeroun, south of Iran and *Cx. pipiens* larvae collected from Tehran, capital of Iran to temephos insecticides was carried out. LC_50_ values of both species were the same that is similar to our findings. Mosquito larvae in all the three geographical regions mentioned above have become resistant to temephos (9). In other countries were reported resistance of adult and larval stages of *Cx. quinquefasciatus* to different groups of insecticides and larvicides. In a study conducted in Morocco, bioassay results showed that *Cx. pipiens* is resistant to temephos that is consistent with our results (36). In Kuala Lumpur (Malaysia), *Cx. quinquefasciatus* larvae were found to be highly resistant to Malathion that was similar to the adults (35). In Central Tunisia, resistance to temephos in *Cx. quinquefasciatus* larvae have been reported (37) Moreover, resistance of *Cx. quinquefasciatus* larvae to malathion, permethrin, and resmethrin has been reported in Florida (USA) (38).

The use of pesticides in agriculture could play a role in the development of resistance to insecticides as well as larvicides in *Cx. quinquefasciatus* in Rafsanjan County (9, 11).

Owing to the emergence of *Cx. quinquefasciatus* resistance to different classes of insecticides and larvicides, using some biological control agents such as *Bacillus thuringiensis* (a Gram-positive, soil-dwelling bacterium) and *Gambusia affinis* (larvivorous fish) can provide an efficient control strategy (39–41). Moreover, use of natural products derived from some plants such as *Bunium persicum* and *Zhumeria majdae* that have no adverse effects on the environment and humans can be suitable and alternative control approach for larvae as well as adult *Cx. quinquefasciatus* mosquitos (42–46).

## Conclusion

Resistance to all tested insecticides was found. The high resistance status observed in the study area may be due to irregular use of pesticides in agriculture led to the constant exposure of the mosquito species to organic chemicals and subsequent development of resistance to insecticides and larvicides in *Cx. quinquefasciatus.* Therefore, regular monitoring of resistance status by standard bioassay and other complementary methods is necessary for the success of future chemical control programs.
